# Costunolide is a dual inhibitor of MEK1 and AKT1/2 that overcomes osimertinib resistance in lung cancer

**DOI:** 10.1186/s12943-022-01662-1

**Published:** 2022-10-06

**Authors:** Xueli Tian, Rui Wang, Tingxuan Gu, Fayang Ma, Kyle Vaughn Laster, Xiang Li, Kangdong Liu, Mee-Hyun Lee, Zigang Dong

**Affiliations:** 1 Department of Pathophysiology, School of Basic Medical Sciences, Zhengzhou University, 450001 Zhengzhou, Henan China; 2 China-US (Henan) Hormel Cancer Institute, No.127, Dongming Road, Jinshui District, 450008 Zhengzhou, Henan China; 3 College of Korean Medicine, Dongshin University, 582 45 Naju, Jeonnam Republic of Korea

**Keywords:** Osimertinib resistance, MEK1, AKT1/2, Costunolide, Combination therapy

## Abstract

EGFR-TKI targeted therapy is one of the most effective treatments for lung cancer patients harboring EGFR activating mutations. However, inhibition response is easily attenuated by drug resistance, which is mainly due to bypass activation or downstream activation. Herein, we established osimertinib-resistant cells by stepwise dose-escalation *in vitro* and an osimertinib-resistant patient-derived xenograft model through persistent treatment *in vivo.* Phosphorylated proteomics identified that MEK1 and AKT1/2 were abnormally activated in resistant cells compared with parental cells. Likewise, EGFR inhibition by osimertinib induced activation of MEK1 and AKT1/2, which weakened osimertinib sensitivity in NSCLC cells. Consequently, this study aimed to identify a novel inhibitor which could suppress resistant cell growth by dual targeting of MEK1 and AKT1/2. Based on computational screening, we identified that costunolide could interact with MEK1 and AKT1/2. Further exploration using *in vitro* kinase assays validated that costunolide inhibited the kinase activity of MEK1 and AKT1/2, which restrained downstream ERK-RSK2 and GSK3β signal transduction and significantly induced cell apoptosis. Remarkably, the combination of osimertinib and costunolide showed synergistic or additive inhibitory effects on tumor growth in osimertinib-resistant cell lines and PDX model. Hence, this study highlights a potential therapeutic strategy for osimertinib-resistant patients through targeting of MEK1 and AKT1/2 by costunolide.

**Supplementary Information:**

The online version contains supplementary material available at 10.1186/s12943-022-01662-1.

## Background

Based on the Global Cancer Statistics of 2020, lung cancer ranks the second most frequently diagnosed cancer (11.4% of total cases) and is the leading cause of cancer-related death (18% of total cancer deaths)[1]. At present, therapeutic regimens for lung cancer include surgery, chemotherapy, immunotherapy, and targeted therapy[2]. Despite the continuous refinement of treatment options, the 5-year survival rate still remains below 20%[3]. Therefore, further investigation is needed to optimize therapeutic strategies.

Of the diverse therapeutic schemes, targeted therapy showed significant preponderance with lower side effects, stronger pertinence, and more convenience for patients[4]. Epidermal Growth Factor Receptor (EGFR)-focused targeted therapy is one of the most widely used treatments for non-small cell lung cancer (NSCLC) patients that harbor EGFR mutations, with more than 60% object response rate[5]. Osimertinib is a third generation EGFR- tyrosine kinase inhibitor (TKI) that has been approved by the FDA as a second-line treatment of EGFR acquired mutant(T790M) NSCLC patient, a first-line treatment for EGFR activating mutant (L858R or exon 19 deletion) NSCLC patients, and as a postoperative adjuvant therapy approved by National Medical Products Administration in China[6]. However, drug resistance is an inevitable issue. Due to tumor heterogeneity, mechanisms of drug resistance vary among different populations and are mainly caused by acquired EGFR mutations, activation or tetraploidization of bypass signal molecules, or phenotypic transformation[5]. Bypass activation, such as Erb-B2 receptor tyrosine kinase 2 (HER2) activation could abnormally activate the mitogen-activated protein kinase (MAPK) or protein-serine-threonine kinase- glycogen synthase kinase 3 beta (AKT-GSK3β) pathways, leading to increased cell proliferation and drug resistance[7]. Currently, EGFR-TKI combined with other drugs are popular regimens for managing drug resistance.

To further explore strategies that could overcome osimertinib resistance, we established osimertinib-resistant cells through a stepwise dose-escalation method and performed phosphorylated proteomics analysis to identify the aberrant activated pathways in resistant cells. In the present study, we identified that mitogen-activated protein kinase kinase 1 (MEK1) and AKT1/2 were abnormally activated in resistant cells. Knockdown of MEK1 and AKT1/2 inhibited the growth of osimertinib-resistant cells and partially restored osimertinib sensitivity. Moreover, we found that costunolide functions as a dual inhibitor of MEK1 and AKT1/2 that significantly induces cell apoptosis in the osimertinib-resistant cell pool. Combination of costunolide with osimertinib showed synergistic or additive inhibitory effect on osimertinib-resistant cells and a resistant patient-derived xenograft (PDX) model. These data demonstrated that costunolide may be considered as a promising strategy for osimertinib-resistant patients with activated MEK1 and AKT1/2.

## Results

### MEK1 and AKT1/2 contribute to the development of osimertinib resistance

**Fig. 1:**
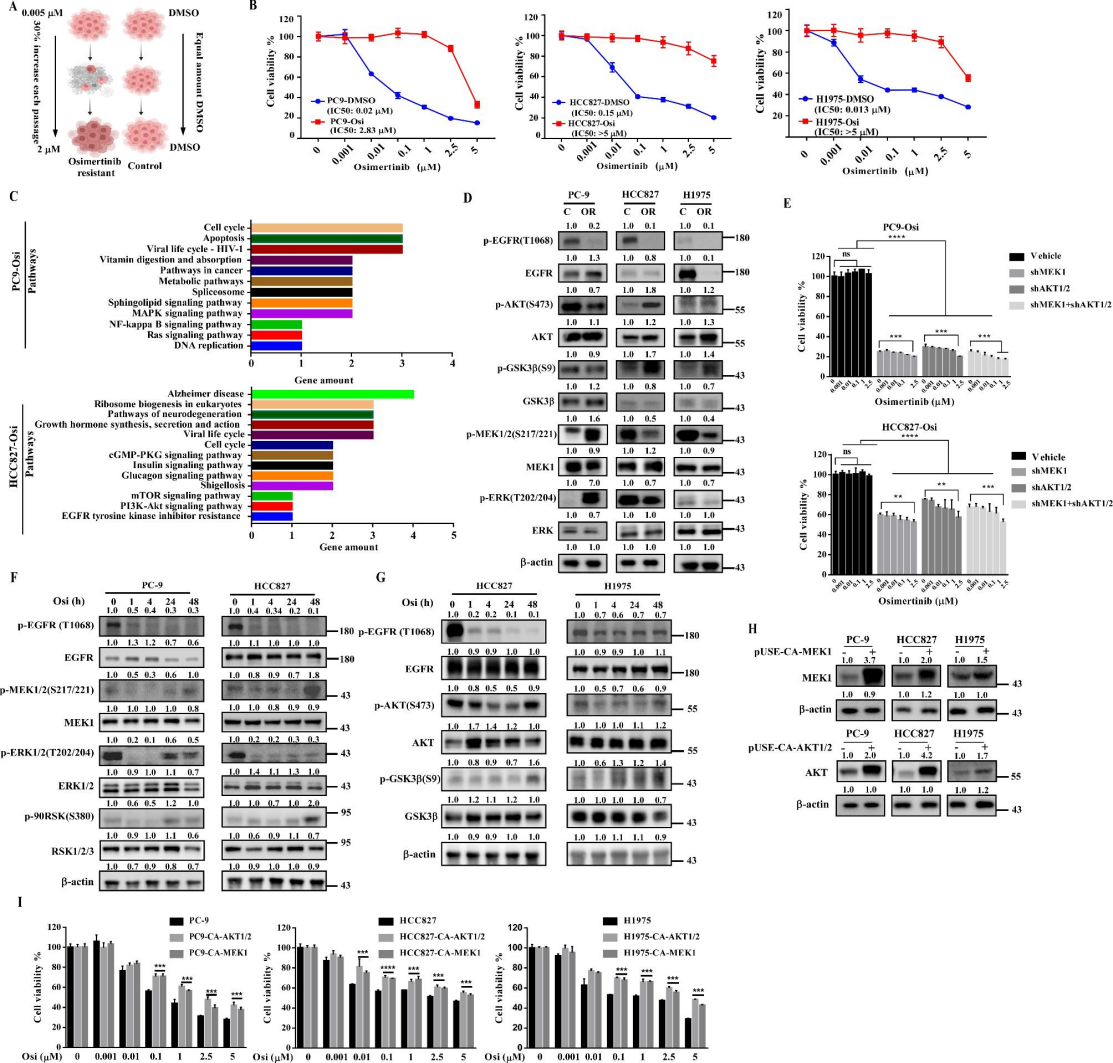
**MEK1 and AKT1/2 drive osimertinib resistance. A**. Method to establish osimertinib resistant cell lines. **B**. Verification of osimertinib sensitivity in parental cells and resistant cells by MTT assay. Both parental cells and resistant cells of PC-9, HCC827 and H1975 were exposed to osimertinib at 0, 0.001, 0.01, 0.1, 1, 2.5,5 µM concentrations for 48 h. Normalized cell viability is shown on the Y axis. IC50 values were calculated using GraphPad Prism 7.0. **C**. Enriched phosphoproteins in resistant cells were analyzed by KEGG. **D**. Cell lysates from resistant cells and parental cells were loaded to compare phosphorylation of MEK1/2 and downstream ERK as well as AKT/ GSK3β. **E**. Cell sensitivity to osimertinib after knockdown of MEK1 and AKT1/2 or dual knockdown of MEK1 and AKT1/2 in PC9-Osi and HCC827-Osi cells. After knockdown, cells were treated with osimertinib at 0, 0.001, 0.01, 0.1, 1, 2.5 µM concentrations for 48 h and cell viability were measured by MTT assay. **F**. Changes in MEK1, ERK and RSK expression by 200 nM osimertinib treatment at the indicated times in PC-9 and HCC827 cells. **G**. Phosphorylation of AKT and GSK3β in HCC827 and H1975 cells treated with 200 nM osimertinib for 1-48 h. **H**. Over-expression of MEK1 and AKT1/2 in PC-9, HCC827 and H1975 cells. pUSE-CA-MEK1 and pUSE -CA-AKT1/2 were transfected into PC-9, HCC827 and H1975 cells; after 24 h, cell lysates were collected to detect expression level of MEK1 and AKT by Western blotting. **I**. Osimertinib sensitivity in control cells and in cells over-expressing MEK1 or AKT1/2. Twenty-four hours after transfection with pUSE-CA-MEK1 and pUSE -CA-AKT1/2, cells were seeded and treated with various concentration of osimertinib for another 48 h. Cell viability was then measured by MTT assay. Quantitative analysis of western blotting bands was performed by Image J software in (D, E, F, G and H). Bars indicate the mean ± SD from 3 independent experiments in (B, E and I). One-way ANOVA with a multiple comparisons test and unpaired t-test were used in (E and I), ns P > 0.05, **P < 0.01, ***P < 0.001, ****P < 0.0001

To further investigate the resistance mechanism of osimertinib and explore the therapeutic regimen accordingly, we established osimertinib-resistant cell lines through a stepwise dose-escalation method (Fig. 1 A). Parental cells treated with DMSO were named as PC9-DMSO, HCC827-DMSO and H1975-DMSO; resistant cells treated with increasing concentration of osimertinib were named as PC9-Osi, HCC827-Osi and H1975-Osi. Next, MTT assays were performed to verify osimertinib sensitivity among parental cells and drug resistant cells. Results indicated that resistant cells showed significant less sensitivity to osimertinib treatment with a higher IC_50_ (Fig. 1B). Additionally, foci formation assays were carried out to assess cell sensitivity to osimertinib treatment. Results showed that osimertinib inhibited foci formation in a dose-dependent manner in parental cells but not in resistant cells (Fig. S1A). To investigate the difference of osimertinib-induced apoptosis in sensitive and resistant cell populations, we measured Annexin V expression after 24 h treatment with various concentrations of osimertinib and found a significantly higher level of apoptosis in parental cells compared with resistant cells (Fig. S1B). Next, phosphoproteomic analysis was performed to verify aberrant signals in parental and resistant cells. Enriched upregulated molecules in resistant cells were analyzed with KEGG analysis. Several signaling pathways were enriched in osimertinib resistant cells. Among them we have narrow-downed against mechanisms of EGFR-TKI resistance and noticed that MAPK and Ras signaling pathways were up-regulated in PC9-Osi cells and PI3K-AKT signaling pathway and EGFR-TKI resistance molecules were up-regulated in HCC827-Osi (Fig. 1 C). Western blot experiments confirmed that MEK/ ERK were significantly activated and AKT/ GSK3β were partially activated in PC9-Osi cells. Likewise, AKT/GSK3β were the dominant activated signaling, but not MEK/ ERK, in HCC827-Osi and H1975-Osi cells (Fig. 1D). The key differences in abnormal signaling between PC9-Osi and HCC827-Osi or H1975-Osi may be attributed to tumor heterogeneity or regulated by alternate activation of upstream signaling proteins. According to previous studies, we measured the expression of p-HER3 (Y1288) and p-HER2 (Y877)[8, 9]. Results indicated that phosphorylation of HER3 is increased in PC9-Osi cells but not in HCC827-Osi or H1975-Osi cells compared with their parental cells (Fig.S1C). When MEK1 and AKT1/2 were knocked down in PC9-Osi and HCC827-Osi, the growth of resistance cells was dramatically inhibited and those cells were partially re-sensitized to osimertinib treatment (Fig. 1E). Hence, our data demonstrated that the MEK1 and AKT1/2 activation were contributors to osimertinib resistance.

To further address the roles of MEK and AKT contributing to osimertinib resistance, we elucidated the temporal process of MEK and AKT activation by treating cells with 200 nM osimertinib for indicated time points. Based on the data, we noticed that downstream effectors of MEK and AKT were inhibited by osimertinib during the short time treatment; however, the signals were subsequently reactivated over prolonged osimertinib treatment (Fig. 1 F, G). This observation suggested that the accumulation of activated MEK and AKT contributed to the osimertinib resistance process. Therefore, we transfected plasmids encoding MEK1 and AKT1/2 into PC-9, HCC827 and H1975 (Fig. 1 H). As shown in Fig. 1I, over-expression of MEK1 and AKT1/2 dramatically decreased osimertinib sensitivity compared with control cells. These findings illustrated that EGFR inhibition facilitated the activation of MEK1 and AKT1/2, thereby driving the osimertinib resistance process. In the case of EGFR-resistance, the choice of targeting one pathway or the other might depends on the types of resistance mechanisms while minimizing drug side effects. For instance, in the case of PI3K-mut, AKT-inhibition is sufficient to abrogate resistance; or in the case of c-MET-amplification, crizotinib or tepotinib are the most effective and currently used pharmacological options. In the case of both MEK1 and AKT1/2 are abnormally activated in osimertinib resistant cells, inhibition of MEK1 and AKT1/2 simultaneously may prevent the further resistance. Thus, identification of a dual inhibitor of MEK1 and AKT1/2 could be paramount for managing osimertinib resistance.

### Costunolide is a novel dual inhibitor of MEK1and AKT1/2 that overcomes osimertinib resistance both in vitro and in vivo

**Fig. 2:**
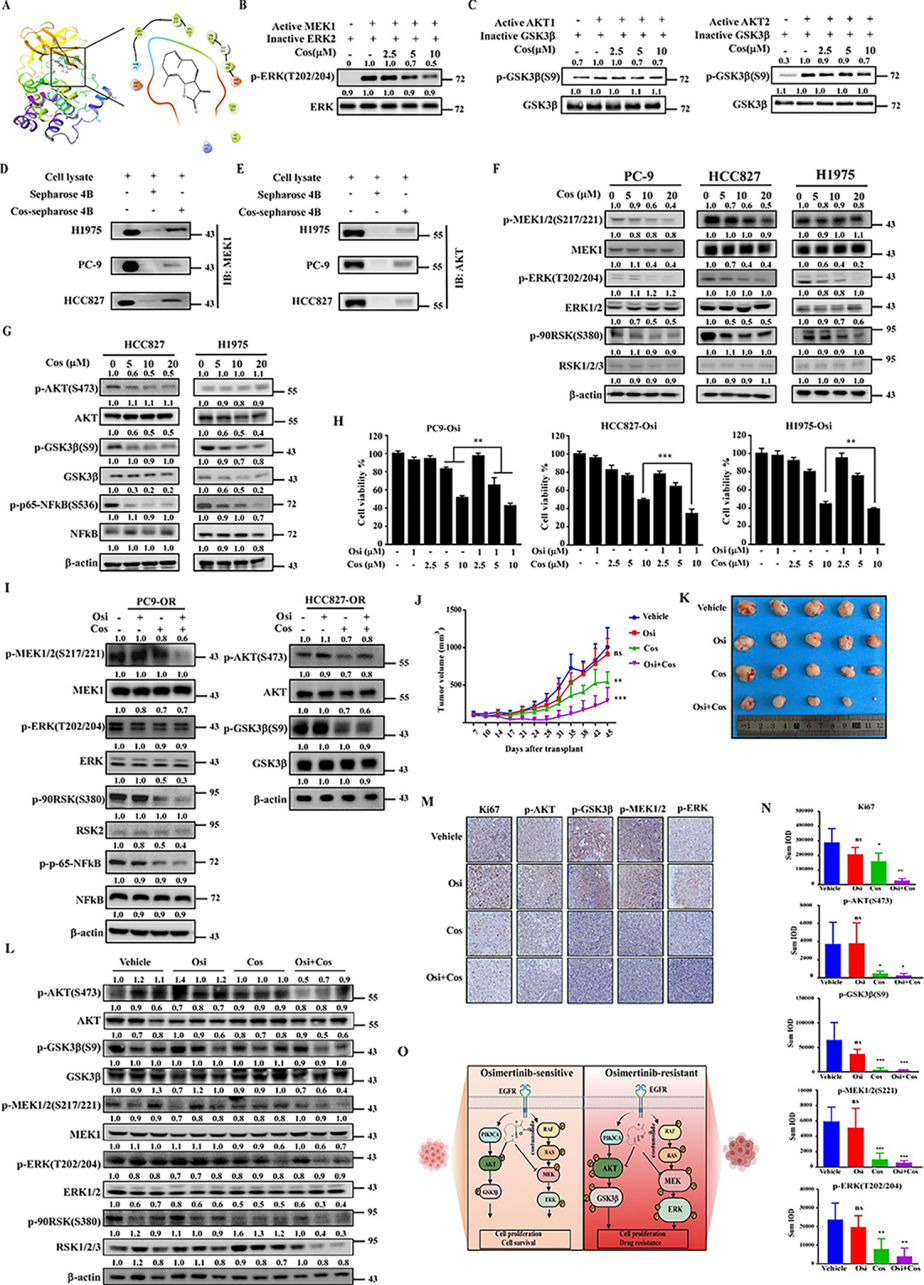
**Costunolide is a dual inhibitor of MEK1 and AKT1/2 that overcomes osimertinib resistance**. **A**. Model of costunolide binding with MEK1. Left: Predicted binding between costunolide and MEK1. Right: Ligand Interaction Diagram (LID) of the binding. MEK1 structure is shown as a ribbon representation and costunolide is shown as a stick. **B**. Inhibitory effect of costunolide on MEK1. 100 ng active MEK1 kinase was pre-incubated with various concentrations of costunolide at RT for 15 min. Next, 200 ng inactive ERK2 and ATP buffer were added and the mixture was incubated at 30 ℃ for 30 min. Phosphorylated and total ERK were detected by Western blot. **C**. The same method was used to confirm the inhibition effect of costunolide on AKT1 and AKT2. Inactive GSK3β was used as the substrate. **D**. Binding between costunolide and MEK1.0.5 mg cell lysates from H1975, PC-9, and HCC827 were incubated with Sepharose-4B or costunolide-conjugated Sepharose-4B. The pulled down proteins were detected by Western blotting. **E**. The same method was used to analyze the binding between costunilode with AKT (D). **F**. Influence of costunolide on MEK and its downstream effectors. PC-9, HCC827, and H1975 cells were treated with costunolide at 0, 5, 10 and 20 µM concentrations for 6 h. Next, the cell lysates were loaded to detect phosphorylation of MEK1/2, ERK, and RSK2. **G**. HCC827 and H1975 cell lysates were used to determine phosphorylation of AKT and downstream GSK3β and NFκB. **H**. Costunolide enhanced the inhibitory effect of osimertinib in PC9-Osi, HCC827-Osi and H1975-Osi cells. Cells were treated with the indicated concentrations of costunolide and osimertinib or in combination for 48 h. Next, cell viability was measured using MTT assays. **I**. Alteration of MEK or AKT signaling. PC9-Osi cells were treated with 1 µM osimertinib, 10 µM costunolide or their combination. Cells were harvested 12 h later and lysed to detect the phosphorylation levels of MEK1, ERK and RSK2. HCC827-Osi cells were treated by costunolide, osimertinib or their combination. After treatment for 12 h, cells were harvested to determine phosphorylation of AKT and GSK3β. **J**. Effect of costunolide (20 mg/kg), osimertinib (10 mg/kg) and their combination on tumor growth. The osimertinib resistance PDX model, HLG57-OR, was inoculated into 4 groups. Drugs were orally administrated every day starting 1 week after transplantation. Tumor volume (length × width× height × 0.52) was measured twice per week. (n = 5) **K**. Image of tumor excised from PDX models. After mice were sacrificed, tumors were excised exfoliated from subcutaneous tissue. **L**. Protein expression level of MEK and AKT, as well as their downstream signaling effectors, in tumor tissues. A portion of each tumor tissue sample was ultrasonicated. 3 samples from each group were loaded to check labeled signaling proteins by Western blotting. **M**. Representative images of ki67, p-MEK1, p-ERK, p-AKT, p-GSK3β expression in tumor tissues. Tissue slides were stained with antibodies and analyzed using IHC. **N.** Expression of ki67, p-AKT1/2, p-GSK3β, p-MEK1, p-ERK in each group. IHC images in each group were analyzed with Image J and total IOD from each image was recorded to indicate the protein expression level. (n = 5) **O**. Diagram illustrating the functional mechanism of costunolide. Quantitative analysis of western blotting bands was calculated by Image J software in (B, C, F, G, I and L). Data was presented as mean ± SD with 3 independent experiments in (H). One-way ANOVA with a multiple comparisons test was applied in (H and N), unpaired t-test was used in (J). ns P > 0.05, **P < 0.01, ***P < 0.001

To identify dual inhibitor of MEK1 and AKT1/2, we performed computational molecular docking using various natural compounds based on the structure of MEK1 and AKT1/2. We identified costunolide could bind with MEK1 (Fig. 2 A). Costunolide is a well-known sesquiterpene lactone that was first extracted from costus (Saussurea lappa Clarke) root. It possesses a 10,5-ring structure with a monocarboxylic acid having three double bonds and two chiral carbons (3aS,11aR). Based on the top docking pose, the binding between MEK1 and costunolide was mediated by hydrophobic interactions surrounding the larger ring, alone with charged interactions between MEK1 ASP190/ASP208 and the γ-lactone ring moiety. The structure of costunolide is illustrated in Fig.S2A. Our previous work showed that coustunolide is an AKT1/2 inhibitor[10]. We hypothesized that costunolide may act as a dual inhibitor of MEK1 and AKT1/2. To test our hypothesis, an *in vitro* kinase assay was performed to verify the inhibitory effect of costumolide on the kinase activity of MEK1 and AKT1/2. Results revealed that costunolide could suppress the kinase activity of both MEK1 and AKT1/2 (Fig. 2B, C); however, costunolide did not decrease the activity of MEK2 (Fig. S2B). To further confirm the results above, we conjugated costunolide with Sepharose 4B beads and conducted an ex vivo pull-down assay using recombinant proteins. Results illustrated that both MEK1 and AKT1/2 recombinant proteins bound with costunolide-conjugated Sepharose 4B but not DMSO (Fig. S3A, B). Additionally, similar binding was observed between costunolide and MEK1 or AKT1/2 from NSCLC cell lysates (Fig. 2D, E). Furthermore, we used pull-down assay to investigate whether costunolide binds to other kinases, including c-Kit, Aurora A, ERK1, ERK2, MKK3, MKK6, RSK2, and PI3K kinases. As shown in Fig S3C, costunolide did not bind with any of the listed kinases. We also examined the effect of costunolide on MEK1, AKT1/2, and other downstream signaling proteins. PC-9, HCC827, H1975 cells were treated with various concentrations of costunolide for 6 h and the protein expression levels of downstream signaling proteins were determined by Western blotting. Results indicated that costunolide suppressed the phosphorylation of MEK, ERK and RSK2 in a dose-dependent manner (Fig. 2 F). Phosphorylation of AKT, GSK3β and NFκB were also inhibited by costunolide (Fig. 2G). Meanwhile, we performed a pull-down assay to assess whether costunolide could directly bind with GSK3β or NFκB; however, our results indicated that costunolide could not bind with GSK3β or NFκB directly (Fig.S3D). Taken together, our findings clearly indicated that costunolide was a dual inhibitor of MEK1 and AKT1/2.

To explore the inhibitory effect of costunolide on cell growth, we performed MTT assays to compare cell viability between parental cells and osimertinib-resistant cells upon treatment with costunolide. Results demonstrated that costunolide have a stronger inhibitory effect on resistant cells compared with parental cells (Fig. S4A). Next, Annexin V staining was measured via flow cytometry to quantify cell apoptosis induced by costunolide treatment. As shown in Fig. S4B, costunolide induced significant cell apoptosis in osimertinib-resistant cells compared to parental cells after 24 h treatment with costunolide. The expression level of cell apoptosis markers, including cleaved PARP, cleaved caspase 7 and cleaved caspase 3 were consistent with the flow cytometry findings (Fig. S4C).

To explore whether costunolide treatment could reverse osimertinib resistance, we examined its toxicity in a normal bronchial epithelial cell line (NL20), an immortalized esophageal cell line (SHEE), and a normal colon cell line (CCD-18Co). As shown in Fig. S5A, costunolide exhibited no cytotoxic effects; 20 µM was taken as the highest concentration for the following studies. We then measured the combinational effect of costunolide and osimertinib on resistant cells. The experimental findings indicated that combination treatment resulted in a significantly reduction in cell viability compared with either osimertinib or costunolide single treatment (Fig. 2 H). Meanwhile, binimetinib (MEK inhibitor) and afuresertib (AKT inhibitor) were selected as positive controls to compare with costunolide. As shown in Fig. S5B, costunolide displayed a stronger inhibitory effect than individual treatment with MEKi and AKTi alone and an even more remarkable inhibitory effect than the combination of MEKi and AKTi in HCC827-Osi cells. Meanwhile, a cytotoxicity assay revealed that AKTi exhibited toxicity in NL20 cells at concentrations greater than 10 µM (Fig. S5C). The findings above suggest that costunolide is an effective and non-toxic compound.

A foci formation assay was next performed to cross-check the combination effect of costunolide with osimertinib. Based on the colony number, inhibition ratio of osimertinib, costunolide, and their combination groups were compared with non-treatment group (Fig. S6A). Then a Q value was calculated to verify the effect of the combination treatment (see the calculation of Q value in Materials and Methods) [11]. Results showed that the Q values calculated in PC9-Osi, HCC827-Osi and H1975-Osi cells were 1.3, 1.4 and 1.13, respectively. These findings indicated that the combination treatment produced a synergistic effect (Q ≥ 1.15) in PC9-Osi and HCC827-Osi cell lines, and an additive effect (0.85 ≤ Q < 1.15) in H1975-Osi cell line. Together, these data implicated that costunolide reversed osimertinib resistance *in vitro*.

As expected, the phosphorylation of MEK and its downstream effectors, ERK and RSK2, were significantly inhibited by costunolide treatment in PC9-Osi cells. Moreover, a more remarkable reduction of phosphorylation MEK1, RSK2 and NFκB were observed in the combination group of costunolide and osimertinib (Fig. 2I). Similarly, combination treatment with costunolide and osimertinib also inhibited the phosphorylation of GSK3β in HCC827-Osi cells (Fig. 2I). Together, our results illustrated that costunolide is an effective compound to overcome osimertinib resistance through modulating the MEK-ERK and AKT-GSK3β signaling pathways.

To verify the therapeutic potential of the costunolide and osimertinib combination treatment *in vivo*, we performed animal experiments using a NSCLC PDX model. An NSCLC tissue (HLG57) harboring an EGFR activating mutation was selected to establish the osimertinib resistant model by continuous induction (Fig. S7A). Briefly, vehicle or 5 mg/kg osimertinib were orally administered to the PDX model each day. Results indicated that osimertinib inhibited tumor growth in the first passage (Fig. S7B). When the tumor volume reached approximately 1000 mm^3^, mice were sacrificed and the largest tumor from passage one was sub-cultured to the second passage. Subsequently, we did not notice the growth inhibitory effect of osimertinib during passage two, even when concentrations as high as 10 mg/kg daily were administered (Fig. S7C). To verify the observation, the largest tumor from the second passage was further sub-cultured to the third passage. Similarly, tumor size and weight in osimertinib treatment group were greater than those measured in the vehicle group (Fig. S7D), indicating that the osimertinib resistant PDX model was established successfully. Next, we used Western blot to identify the alterations in commonly activated signaling pathways that occur during the osimertinib resistance process. From the results, we observed that the phosphorylation of Her3 was dramatically increased in osimertinib resistant tumor tissues (Fig. S7E). Herein, the control PDX model and osimertinib treated PDX model were named as HLG57-DMSO and HLG57-Osi, respectively.

Tumors from HLG57-DMSO and HLG57-Osi were inoculated into NOD/SCID mice and divided into 4 groups each. Vehicle, osimertinib (10 mg/kg), costunolide (20 mg/kg) and osimertinib plus costunolide were administrated orally each day. Tumor volume and body weight were recorded twice per week. Results indicated that costunolide inhibited HLG57-Osi growth and that the combination treatment showed a more obvious growth inhibitory effect (Fig. 2 J, K, Fig. S8A). Additionally, no significant difference in body weight was observed in mice treated with osimertinib and costunolide relative to the vehicle-treated group (Fig. S8B). Furthermore, we measured the protein expression levels of MEK, AKT and their downstream effectors by Western blot. Results showed that the combination treatment attenuated signal transduction efficiently, with the lowest phosphorylation observed in ERK, RSK2, NFκB and GSK3β (Fig. 2 L). IHC staining produced similar results as those observed in the Western blot analysis (Fig. 2 M, N). In addition, expression of the Ki-67 tumor proliferation marker was mostly decreased in the combination treatment group (Fig. 2 M, N). To confirm the toxicity of costunolide and osimertinib, we checked the morphology of liver, kidney and spleen by H&E staining. No obvious differences were observed among different treatment groups (Fig. S9A, B). Additionally, ALT and AST expression levels in the plasma were not significantly altered compared with vehicle groups (Fig. S9C, D). We also measured the number of WBC and RBC in the blood of mice 24 h after treatment with vehicle or costunolide (20 mg/kg). From the results, we did not notice significant changes in the number of WBC or RBC between the vehicle and costunolide treatment group (Fig. S9E). The observations above suggested that the inhibition of MEK1 and AKT1/2 by treatment with costunolide reversed osimertinib resistance tumor progression through modulation of the ERK/RSK and AKT-GSK3β signaling axis (Fig. 2O).

In the HLG57-DMSO group, we noticed that osimertinib dramatically inhibited tumor growth (Fig. S10A, B), once again confirming a successfully osimertinib resistance model of HLG57-Osi. Similar to our previous data, no significant changes in body weight between groups were observed (Fig. S10C). However, we did not notice a significant growth inhibitory effect of costunolide in HLG57-DMSO (Fig. S10A, B); this observation is mainly due to the low expression of p-MEK and p-AKT in HLG57 tumor tissue compared with other tissues (Fig. S10D). Thus, we suggested that the inhibitory effect of costunolide is dependent on the expression of activated MEK and AKT.

## Discussion

EGFR targeted therapy has achieved prominent performance for NSCLC treatment; however, acquired drug resistance inevitably limits long-term effects[7]. An appropriate drug resistant model is rather important for preclinical studies. Consequently, we generated osmertinib-resistance in cell lines harboring EFGR mutations through a step-wise dose escalation method, which showed a remarkably higher IC_50_ of osimertinib compared with parental cells. The lower drug susceptibility was further confirmed by foci formation and cell apoptosis assays in the resistant cells. To establish a more comprehensive resistance mechanism *in vivo*, we also generated an osimertinib-resistant PDX model through continuous induction using lung cancer tissue harboring an EGFR mutation. These long-term inducted resistant models are effective tools to realistically simulate the process of drug resistance in a laboratory setting.

Due to tumor heterogeneity, the reported mechanisms of osimertinib resistance may vary depending on the terms of different regimens. Acquired EGFR mutation, c-MET amplification, HER2 amplification or mutation, PIK3CA mutation, BRAF and KRAS mutation have been reported as the dominant factors contributing to osimertinib resistance in response to first-line treatment. Acquired EGFR mutation, c-MET amplification, cell cycle gene alteration, HER2 amplification, PIK3CA amplification or mutation have been reported as contributors to osimertinib resistance in response to second-line treatment. Obviously, most of the dysregulated proteins highlighted above can activate PI3K/AKT and MAPK-ERK pathways. As reported, AKT is a key modulator in regulating multi-drug resistance[12]. One mechanism occurs through AKT-triggered activation of NFκB, which can inhibit cell apoptosis and promote tumor growth. Furthermore, activated AKT also modulates cell proliferation through the phosphorylation of GSK3β, which can facilitate resistance by promoting the evasion of EGFR-targeted therapy. Besides, MEK also plays a profound role in regulating drug resistance. The paradoxical activation of MEK stimulates ERK to promote cell proliferation and drug resistance[13]. Most often, activation of MEK or AKT also play crucial roles during the drug resistance process. As reported, combination of gefitinib with MEK1/2 inhibitor synergistically inhibited gefitinib-resistant NSCLC cell growth[14]. Dual blockade of PI3K/AKT and MEK/ERK pathways potentiated gefitinib sensitivity in gefitinib resistant NSCLC and breast cancer cells. Accordingly, AKT/GSK and MEK/ERK are the most frequently dysregulated signaling pathways in acquired drug resistance. However, individually targeting AKT or MEK may facilitate active bypass or downstream signaling which will limit the success of therapies. Thus, the rational to inhibit PI3K/AKT and MAPK pathways simultaneously seems logical to produce a more robust inhibitory response that may prevent further resistance. In present study, we identified that costunolide is an effective inhibitor capable of suppressing the kinase activity of MEK1 and AKT1/2, thereby inducing significant cell apoptosis and inhibition of cell growth. Costunolide is a natural bioactive sesquiterpene lactone with antioxidant, anti-inflammatory and anticancer effects that is extracted from the roots of Saussurea lappa. Recent studies have shown that costunolide can inhibit the proliferation of various cancer cells. In ovarian cancer cells, costunolide promotes the expression of apoptosis signals, such as caspase 3, caspase 8 and caspase 9 by enhancing the production of ROS, thereby inhibiting the growth of cisplatin-resistant cells[15]. In addition, costunolide can inhibit the growth of colorectal cancer and melanoma cells by inhibiting the kinase activity of AKT[10]. Costunolide also showed a similar inhibitory effect compared with the combination of AKTi and MEKi, but at a higher dose. Our data suggested that, costunolide could act as a safe and effective inhibitor to suppress osimertinib-resistant cell growth.

Another critical finding of our study is that costunolide reversed osimertinib resistance *in vivo*. Due to the stable biological characteristics of patient derived tissues, we used an EGFR mutant PDX model to further evaluate the combination effects of costunolide and osimertinib. Based on the data, costunolide inhibited tumor growth and a significant synergistic effect was observed in the model. Moreover, downstream signaling effectors of MEK and AKT were markedly inhibited in the combination treatment group. Additionally, we did not observe obvious changes in total body weight, ALT or AST level between the different groups, indicating a well-tolerated dose of costunolide plus osimertinib. However, it should be noted that costunolide did not show a growth inhibitory effect in the HLG57-DMSO model. This observation is mainly because p-MEK and p-AKT protein expression levels are lower in the HLG57 relative to other lung tumor tissues. Based on this *in vivo* study, we concluded that the efficiency of costunolide is dependent on the levels of activated MEK1 and AKT1/2. Additional studies are required to further characterize suitable strategies for managing osimertinib-resistant cell populations deficient in active MEK and AKT.

## Conclusion

Our study demonstrated that MEK1 and AKT1/2 are critical for the development of osimertinib resistance. Moreover, costunolide reversed osimertinib resistance through direct targeting of MEK1 and AKT1/2. A synergistic or additive effect was observed with the combination treatment of costunolide and osimertinb both *in vitro* and *in vivo*, which might offer a candidate strategy in the clinic.

## Electronic supplementary material

Below is the link to the electronic supplementary material.

Supplementary Material 1

Supplementary Material 2

Supplementary Material 3

Supplementary Material 4

Supplementary Material 5

Supplementary Material 6

Supplementary Material 7

Supplementary Material 8

Supplementary Material 9

Supplementary Material 10

Supplementary Material 11

Supplementary Material 12

## Data Availability

All data generated or analyzed in this project are included with this published article and its supplementary information files.

## Abbreviations

EGFR: epidermal growth factor receptor
NSCLC: non-small cell lung cancer
TKI: tyrosine kinase inhibitor
HER2: Erb-B2 receptor tyrosine kinase 2
BRAF: B-Raf Proto-Oncogene
PIK3CA: phosphatidylinositol-4,5-Bisphosphate 3-kinase catalytic subunit alpha
AKT: protein-serine-threonine kinase
GSK3β: glycogen synthase kinase 3 beta
MEK1: mitogen-activated protein kinase kinase 1
PDX: patient-derived xenograft
