# Supplementary material for: Costunolide is a dual inhibitor of MEK1 and AKT1/2 that overcomes osimertinib resistance in lung cancer
Source: Mol Cancer. 2022 Oct 6;21:193. doi: 10.1186/s12943-022-01662-1 (PMC9535870; doi:10.1186/s12943-022-01662-1)
Supplement: Supplementary file 1 — Supplementary Material 1 [file 12943_2022_1662_MOESM1_ESM.docx]

**Materials and methods**

*Reagents*

Osimertinib (purity ≥97%) was purchased from MCE (Wuhan, China). Costunolide (purity ≥98%) was purchased from Wuhan jonk biological technology Co., Ltd (Wuhan, China). Both active kinase (MEK1, MEK2, AKT1, AKT2) and unactive substrates (ERK2 and GSK3β) recombinant proteins for kinase assay were purchased from SignalChem (Richmond, BC). Antibodies against phosphorylated AKT (p-AKT, Ser 473), AKT (pan), phosphorylated glycogen synthase kinase 3 beta (p-GSK3β, Ser 9), GSK3β, cleaved PARP, cleaved Caspase 3, cleaved Caspase 7 were purchased from Cell Signaling Technology (Beverly, MA). Antibodies to detect β-actin was purchased from Zhongshan Jinqiao Biological Technology Co., Ltd (Beijing, China). ALT and AST measure kit were purchased from Nanjing Jiancheng Bioengineering institute (Nanjing, China)

*Cell culture and reagents*

The human NSCLC cell lines H1975, Hcc827, PC-9 were purchased from the Type Culture Collection of the Chinese Academy of Sciences (Shanghai, China). All of them were cultured with RPMI 1640 (VivaCell, shanghai, China) containing 10% FBS (VivaCell, shanghai, China) and 1% Penicillin-Streptomycin (NCM, Suzhou, China). NL20 was cultured in Ham’s F12 mediun (Lonza, Walker, USA) containing 0.324 g/L NaHCO_3_ (Kermel, Tianjin, China), 0.9g/L glucose (Kermel, Tianjin, China), 1 μM L-glutamine, 0.1 μM NEAA (Gibco, NY, USA), 0.005 mg/ml insulin (Solarbio, Beijing, China), 10 ng/ml EGF (PeproTech, NJ, USA), 0.001mg/ml transferrin (Solarbio, Beijing, China), 500 ng/ml hydrocortisone (MCE, Hubei, China), 4% FBS and 1% PS. 2.2 μM osimertinib was additional added in the RPMI 1640 medium for resistant cell maintaining culture.

*Establishment of osimertinib resistant cells*

H1975, PC-9, Hcc827 cells were seeded in 100 mm culture dishes. The next day, an initial dosage of 0.05μM osimertinib was added into the culture medium. When cells density reached to 85%, cells were sub-cultured with 30% increased dosage of osimertinib compared to the last passage. By analogy, modeling process finished until osimertinib reached to 2 μM. Same amount DMSO was treated in the control group.

*Establishment of osimertinib resistant PDX model*

6–7 weeks old female mice (Vital River Labs, Beijing, China) with severe combined immunodeficiency (SCID) were employed to eatablish osimertinib-resistant PDX model following the guidelines established by Zhengzhou University Institutional Animal Care and Use Committee (Zhengzhou, China) under “specific pathogen-free” conditions as previously described. Brifly, tumor tissue harbor EGFR activation mutation was cutted into small pieces with an average amount of 0.1 g. The cutted tissues were implanted into the back of SCID mice pretreated with Penicillin-Streptomycin. Inoculated mice were grouped into osimertinib treatment group and control group. 5mg/kg osimertinib were administered by oral gavage once a day in osimertinib treatment group. Control group were treated with equal vehicle. Tumor volume and body weight were recorded twice per week. When the average of tumor volume reached to about 1000mm^3^, the biggest one or two mice were sacrificed to subculture to the next passage. 10mg/kg osimertinib were administered to osimertinib treatment group from the second passage. Resistant model was established until the tumor volume are bigger in the osimertinib group than that in the control group.

*Cell proliferation assay*

PC-9 and H1975 (1.5 × 10^3^ cells per well) or Hcc827 (3 × 10^3^ cells per well) cells were seeded in 96-well plates with 150 μl complete RPMI-1640 medium and incubated for 24 h at 37 °C in a 5% CO_2_ incubator. Various concentrations of osimertinib or costunolide (dissolved in DMSO) or vehicle (DMSO) in 50 μl of complete growth medium were added to each well to a final volume of 200 μl. Cells were cultured for another 48 h. To evaluate cell proliferation ability, 20 μl MTT solution (Solarbio, Beijing, China) were added to each well. 2h later, culture medium was removed with the help of a vacuum system. Subsequently, 150 μl of DMSO was added to dissolve the crystal. Absorbance at 570 nm was measured using the Thermo Multiskan plate-reader (Thermo Fisher Scientific, Waltham, MA, USA) and cell viability was calculated accordingly.

*Foci assay*

800 cells suspended in 1.5ml culture medium were seeded in each well of a 6-well plate. 24h later, 50 μl series dilutions of osmertinib were added to each well. Cells were maintained at 37 °C in a 5% CO_2_ incubator for 1 week. Then culture medium was removed and colonies were washed with PBS and stained with crystal violet solution. Number of colonies was quantified using the Image-Pro Plus software (v.6) program (Media Cybernetics, Rockville, MD, USA).

*Cell apoptosis assay*

5×10^4^ cells were seeded in a 60 mm culture dish with 3 ml culture medium and incubated at 37 °C in a 5% CO_2_ incubator. 24h later, indicated concentration of osimertinib or costumolide were added into culture medium. Drugs were incubated with cells for 24h. Then all cells including the dead cells in the supernatant were harvested for apoptosis analyze. The harvested cells were washed with cold PBS and then suspended with 100μl binding buffer and subsequently stained with FITC-Annexin V (BioLegend, San Diego, CA) solution and PI solution (Solarbio, Beijing, China). Early and late-stage apoptosis were quantified with FL1 and FL3 channel by flow cytometry.

*Phosphoproteomics assay*

PC9-DMSO, PC9-Osi, Hcc827-DMSO and Hcc827-Osi cells (2×10^7^ cells) were lysised to get 2 mg cell lysate. Briefly, cell lysates were denatured with 8 M urea at 37 ℃ for 1 h. Then lysates were oxidative degraded with 50 mM IAA at RT for 0.5 h. Then the reaction buffer was exchanged with 50 mM NH_4_HCO_3_ buffer. Next, trypsin was added to digest proteins to peptides. Peptides were immobilized and enriched with active TiO2 beads. Finally, phosphorated peptides were eluted with elute buffer 1 and buffer 2 (elute buffer 1: 300 mM ammonia in 50% ACN; elute buffer 2: 500 mM ammonia in 60% ACN). Phosphopeptides were continue processed with mass spectrometry. Enriched signals in resistance cells were analyzed with KEGG (www.kegg.jp).

*Computer Docking*

An in-silico docking assay using the Schrödinger Suite 2015 software programs.29 were used to predict the binding between MEK1 and costunolide. Briefly, crystal structure of MEK1 downloaded from the protein data bank (http://www.rcsb.org/pdb) was further processed with standard procedures of the Protein Preparation Wizard. All water molecules were removed and hydrogen atoms were added consistent with a pH of 7 instead. The ATP-binding site-based receptor grid of MEK1 was generated for docking with costunolide by default parameters using the LigPrep program under the extra precision (XP) mode using the program Glide.

*Pull-down assay*

500 μg cell lysates from PC-9, H1975, Hcc827 were rotated with 50 μl Sepharose 4B-costunolide or Sepharose 4B-DMSO in reaction buffer (50mM Tris pH 7.5, 5mM EDTA, 150mM NaCl, 1mM DTT, 0.01% NP40, 2 μg/ml bovine serum albumin) at 4 °C. After incubation at 4℃ overnight, beads were washed with washing buffer (50mM Tris pH 7.5, 5mM EDTA, 150mM NaCl, 1mM DTT, 0.01% NP40) to completely remove unspecific binding protein. The cleaned beads were denatured with SDS-sample buffer and visualized by Western blotting.

*In vitro kinase assay*

Active MEK1, active MEK2, active AKT1, active AKT2, unactive ERK2, unactive ERK1 and unactive GSK3β were purchased from signal biology. 100 ng active kinase were incubated with indicated concentration of costunolide for 15 min at RT. Then 200 ng unactive substrate and ATP buffer were added and the mixtures were incubated at 30 ℃ for 30 mins. The phosphorylated substrates were indicated through western blot.

*Western blot*

Cell pellet were lysed with suitable volume of RIPA buffer (150 mM NaCl, 1% NP-40, 50 mM Tris–HCl, 1 mM PMSF, 1× protease inhibitor) for 1h on ice. Total protein from the supernatant of cell lysate were quantified using the BCA Quantification Kit (Solarbio, Beijing, China). 5× SDS-sample buffer was used to denature the protein. 30 ug protein were loaded in each well of the SDS-PAGE to detect the phosphate protein, including p-MEK, p-ERK, p-RSK2, p-NFκb, p-AKT, p-GSK3β. 15 ug total protein were loaded to detect corresponding total protein, including MEK, ERK, RSK2, NFκb, AKT, GSK3β. 10ug protein were loaded to detect actin as an internal reference. Proteins were subjected to 10% SDS-PAGE gels electrophoresis and transferred to a PVDF membranes (Millipore, Billerica, MA). After blotting with 5% skim milk, membranes were incubated with primary antibodies against specific targets at 4℃ overnight. The next day, membranes were washed with TBST buffer thoroughly and incubated with proper secondary antibodies. The protein bands were visualized by ECL buffer through the Amersham Image 600 (GE, Milwaukee, WI) imager.

*Hematoxylin and eosin (H&E) staining or immunohistochemistry (IHC) assay*

The liver, spleen, kidney, and tumor tissues from mice were soaked in 4% formaldehyde. Then tissues were dehydrated with different concentration ethanol before embedded in paraffin blocks for H&E staining or IHC. For H&E staining, tissue sections of tumor, liver, kidney and spleen were deparaffinized at 65℃ for 1 h and then stain with hematoxylin and eosin. For IHC assay, tumor tissue sections were deparaffinized at 65℃ for 2 h and then rehydration with the help of. Then slides were incubated with indicated primary antibodies following antigen retrieval with 10mM citrate acid and blocking with 5% BSA. The next day, an HRP-conjugated goat anti-rabbit (ZSGB-BIO, Beijing, China) was added and incubated for 30min. Then tissue sections were developed with DAB solution (ZSGB-BIO) for 10-40 s and the reaction was terminated by water. Finally, sections were redyed with hematoxylin. All sections were observed by microscope and analyzed using the Image-Pro Plus software (v. 6) program.

*Calculation of combination Index*

According to colony number in foci formation assay. Inhibition effects of the individual and combination treatment were calculated by compare with non-treatment group. Then a Q value was calculated according to the inhibition effects. Q=Ea+b/(Ea+Eb-Ea×Eb), Ea+b: inhibition effect of the combination group; Ea: inhibition effect of osimertinib; Eb: inhibition effect of costunolide. Q≥1.15 means synergistic effect; 0.85≤Q<1.15 means additive effect; Q<0.85 means a antagonistic effect.

*In vivo toxicity assay*

Blood samples from each group were collected to mesure glutamic pyruvic transaminase (ALT) and glutamic-oxalacetic transaminase (AST) activity to evaluate the toxicity *in vivo*. First, serum was separated from blood immediately after collection. According to the kit instruction, ALT or AST react with substrates and DNPH and produce colored phenylhydrazone, which was used to indicate ALT and AST activity by detecting the absorbance at 510 nm.

*Statistical analysis*

All quantitative results are expressed as mean ± S.D. or ± S. E values. Significant differences were compared using the student’s t-test with GraphPad Prism 7. Differences with a p < 0.05 were considered to be statistically significant and labeled with (*), p < 0.01 labeled with (**), p < 0.001 labeled with (***), p < 0.0001 labeled with (****).
