# Supplementary figures and images for: Costunolide is a dual inhibitor of MEK1 and AKT1/2 that overcomes osimertinib resistance in lung cancer
Source: Mol Cancer. 2022 Oct 6;21:193. doi: 10.1186/s12943-022-01662-1 (PMC9535870; doi:10.1186/s12943-022-01662-1)

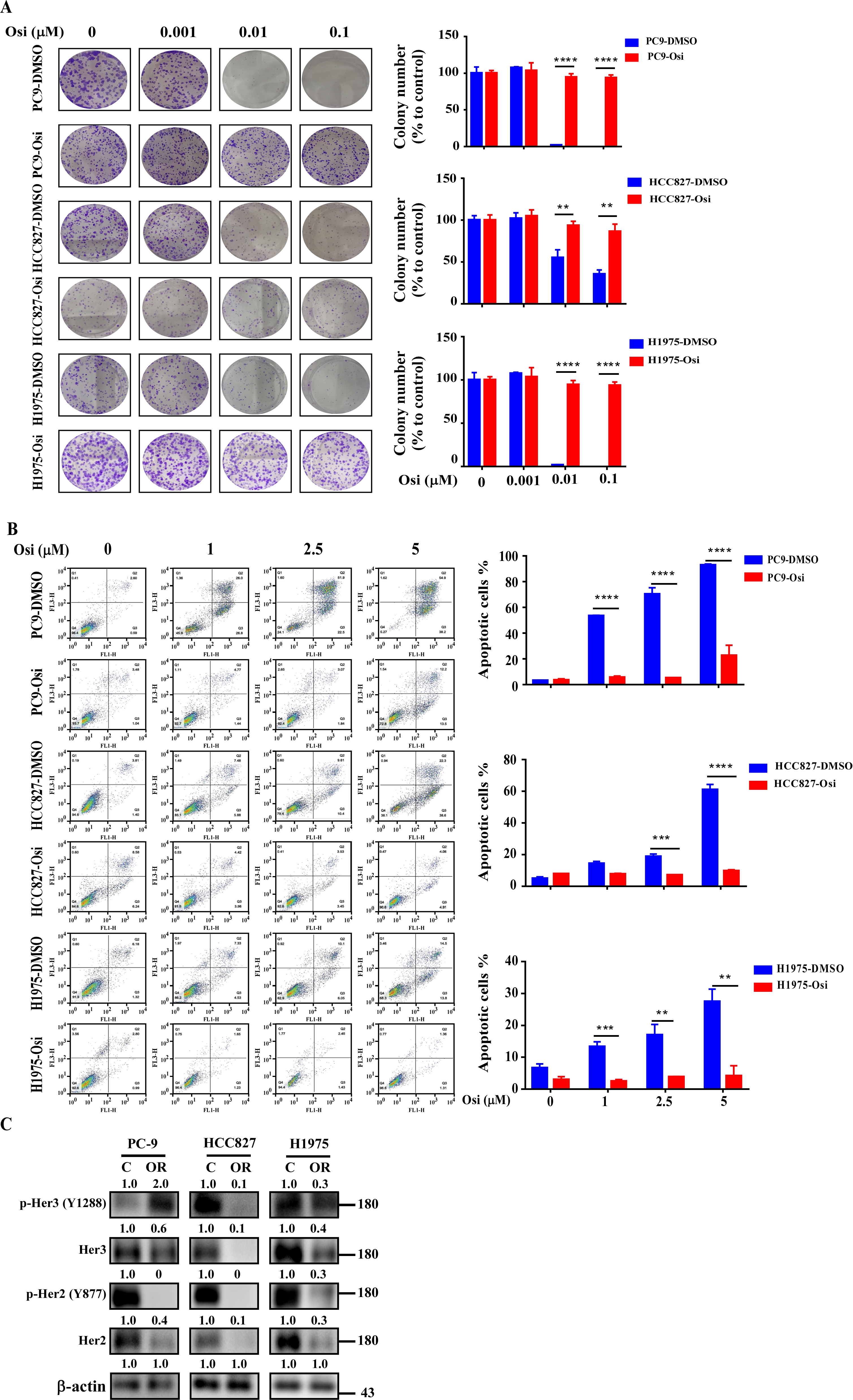

Supplement: Supplementary file 2 — Supplementary Material 2 [file 12943_2022_1662_MOESM2_ESM.jpg]

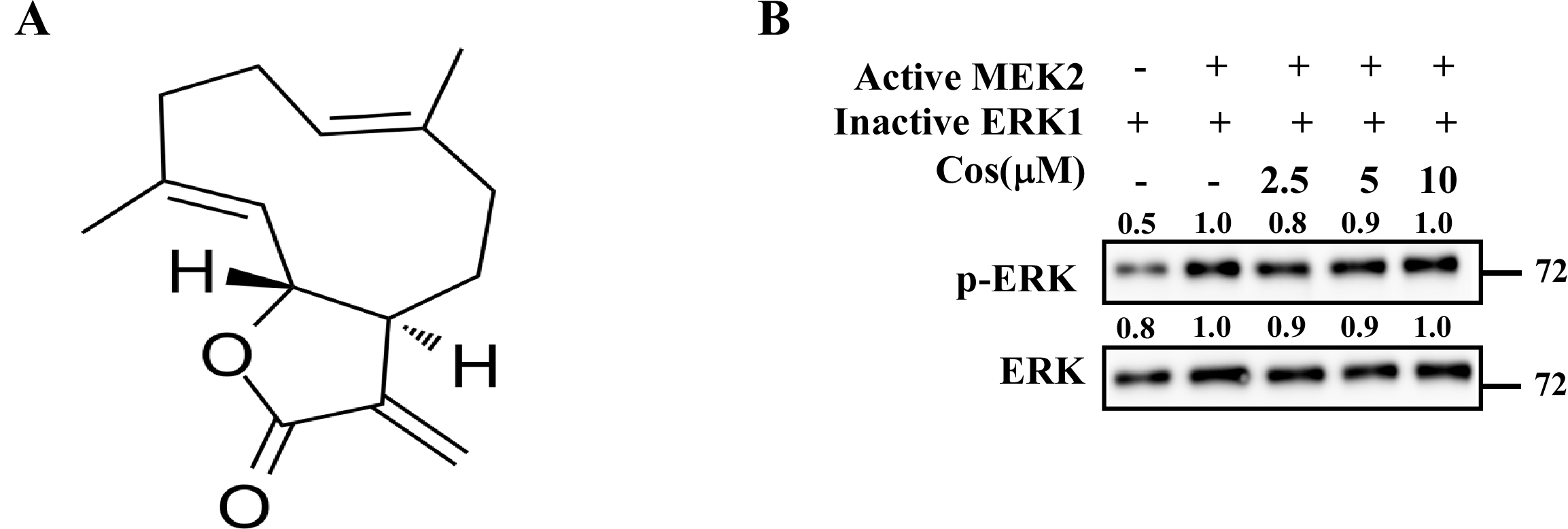

Supplement: Supplementary file 3 — Supplementary Material 3 [file 12943_2022_1662_MOESM3_ESM.jpg]

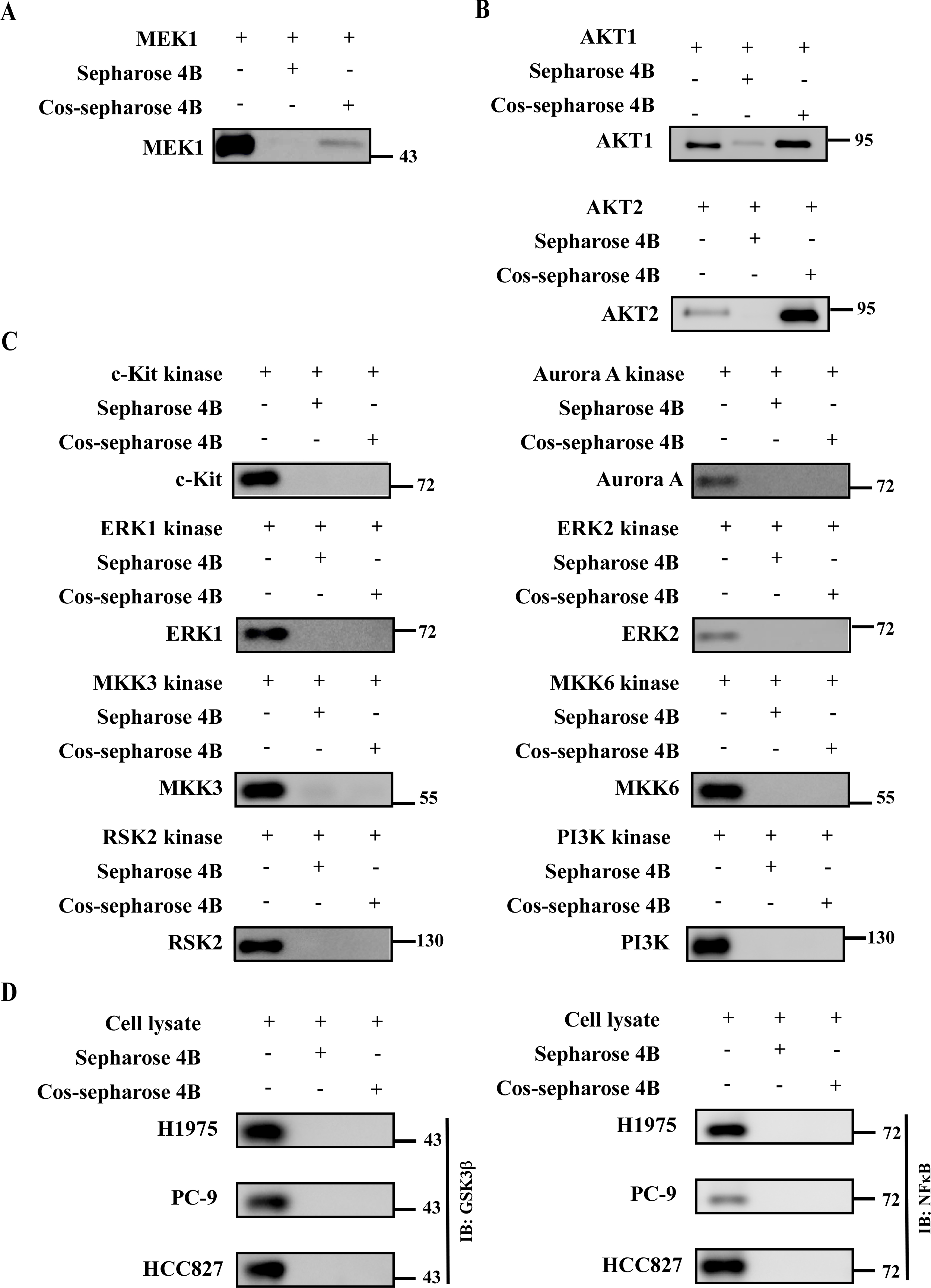

Supplement: Supplementary file 4 — Supplementary Material 4 [file 12943_2022_1662_MOESM4_ESM.jpg]

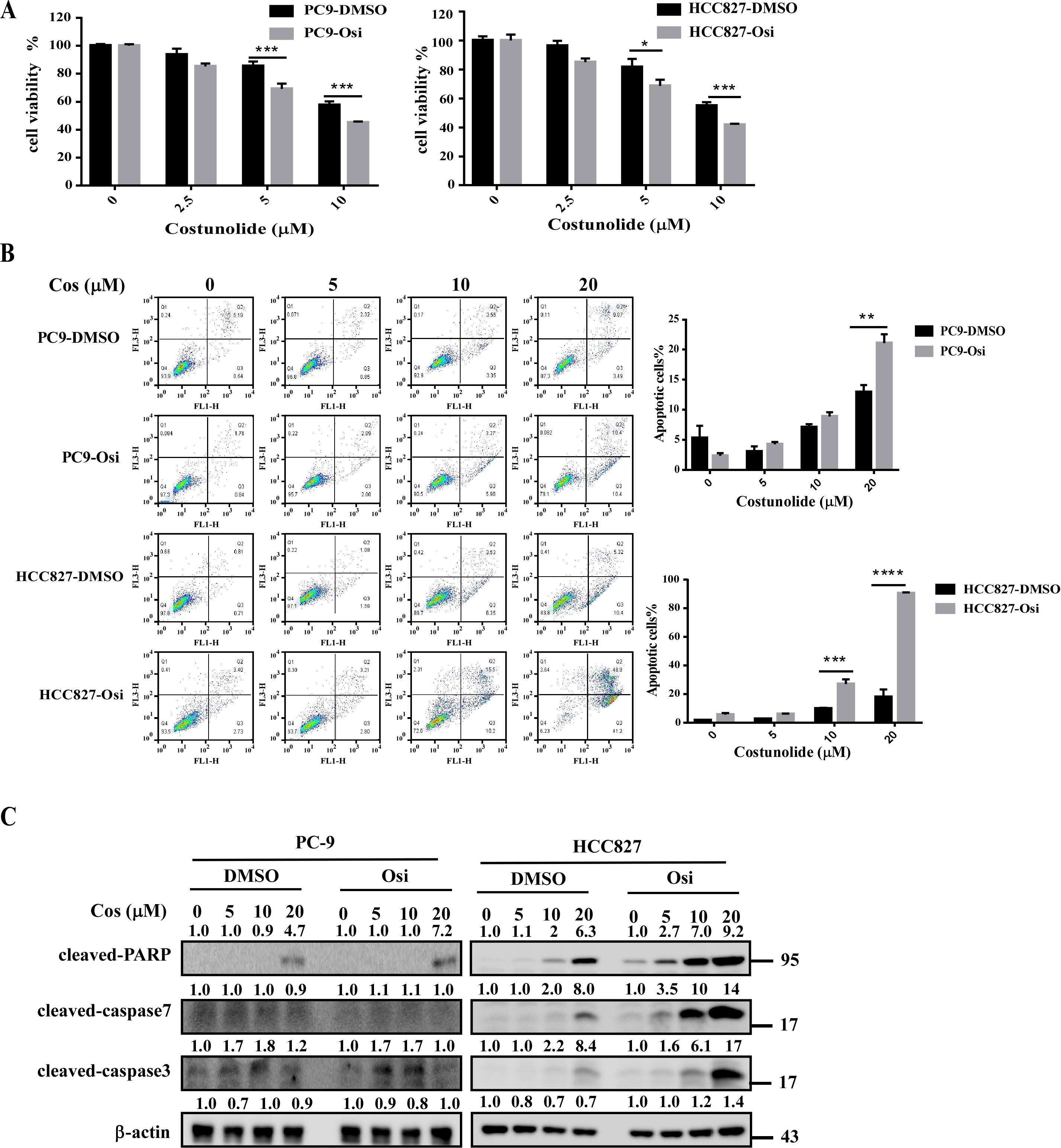

Supplement: Supplementary file 5 — Supplementary Material 5 [file 12943_2022_1662_MOESM5_ESM.jpg]

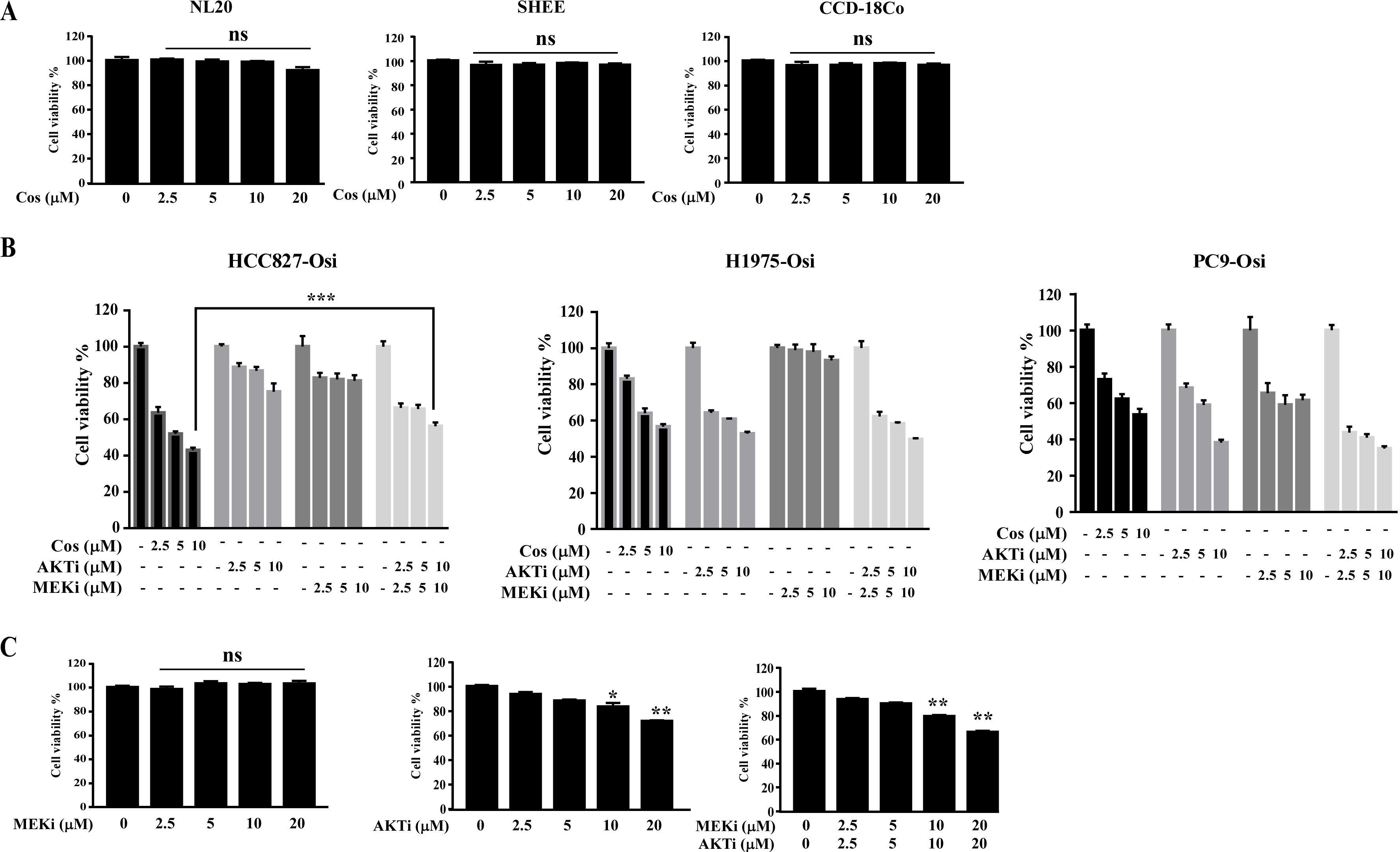

Supplement: Supplementary file 6 — Supplementary Material 6 [file 12943_2022_1662_MOESM6_ESM.jpg]

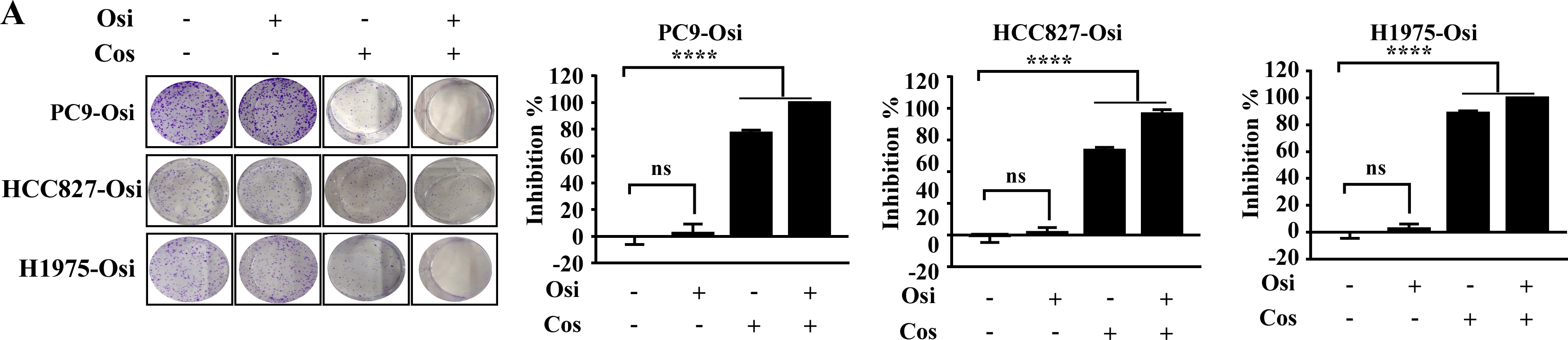

Supplement: Supplementary file 7 — Supplementary Material 7 [file 12943_2022_1662_MOESM7_ESM.jpg]

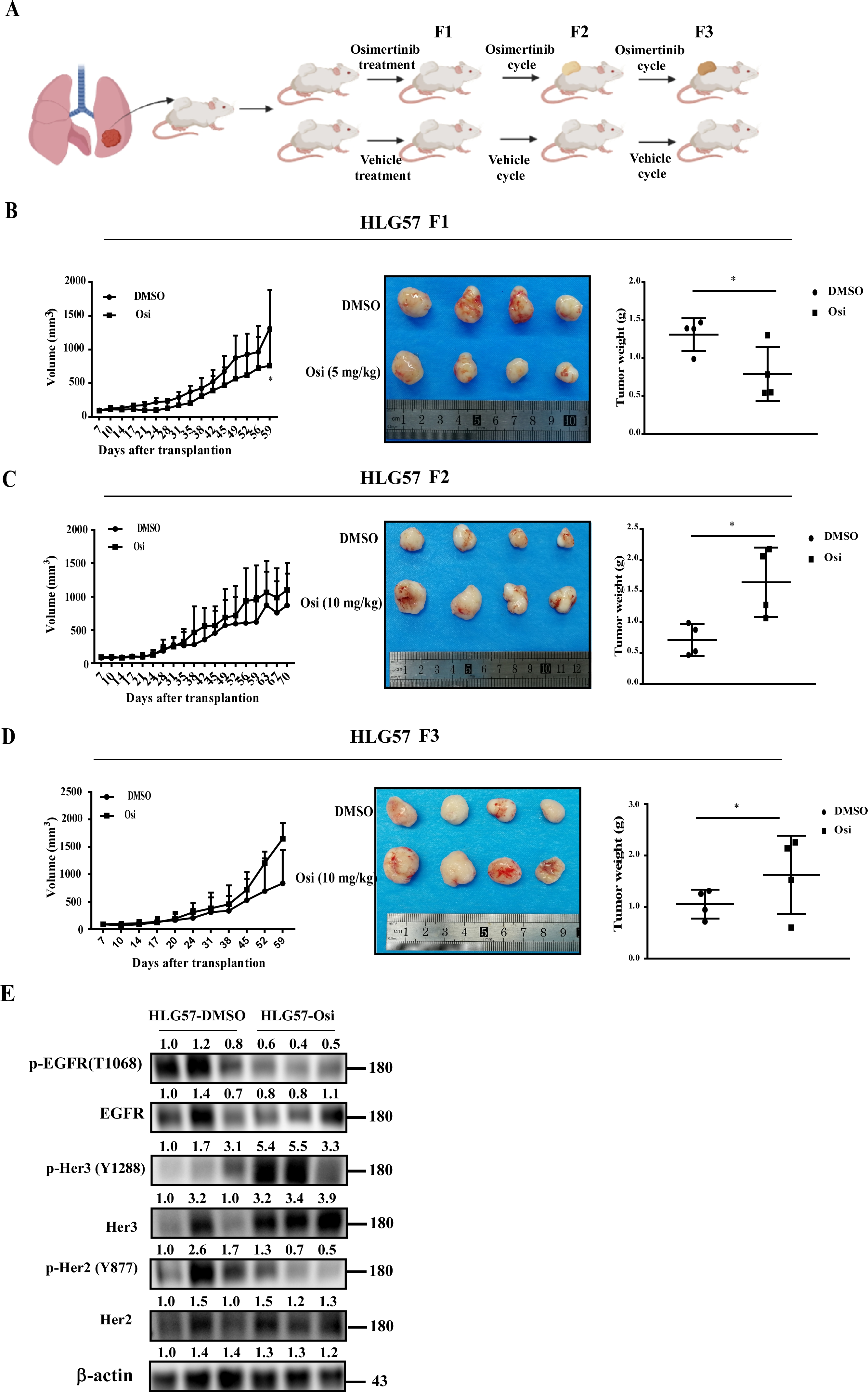

Supplement: Supplementary file 8 — Supplementary Material 8 [file 12943_2022_1662_MOESM8_ESM.jpg]

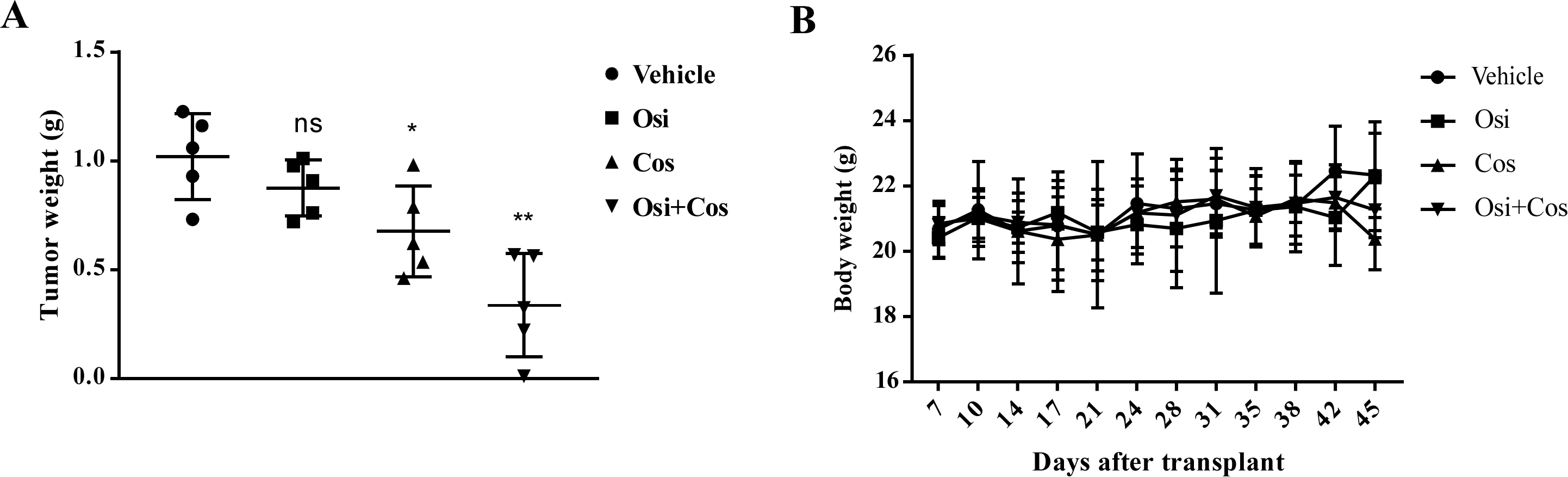

Supplement: Supplementary file 9 — Supplementary Material 9 [file 12943_2022_1662_MOESM9_ESM.jpg]

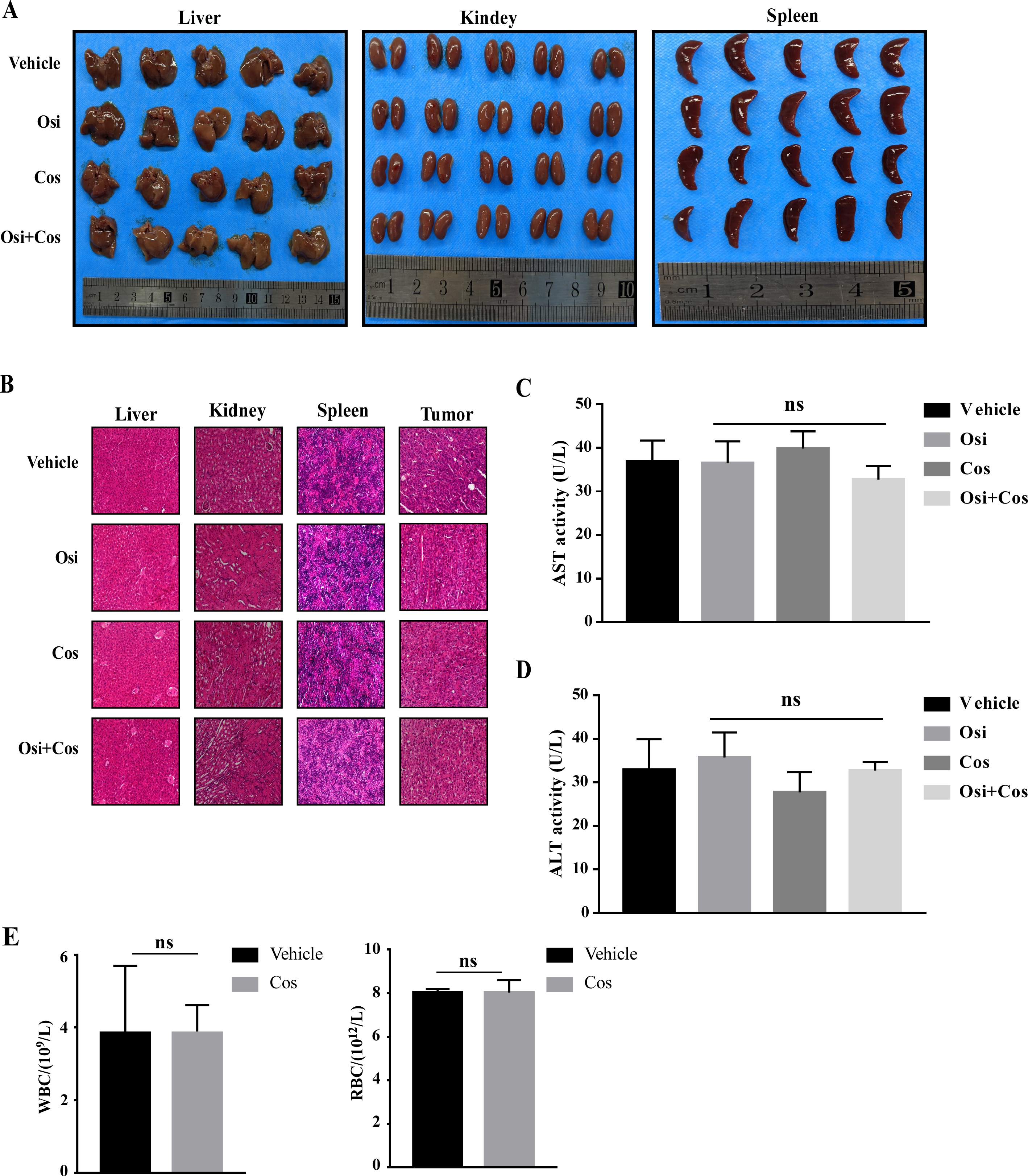

Supplement: Supplementary file 10 — Supplementary Material 10 [file 12943_2022_1662_MOESM10_ESM.jpg]

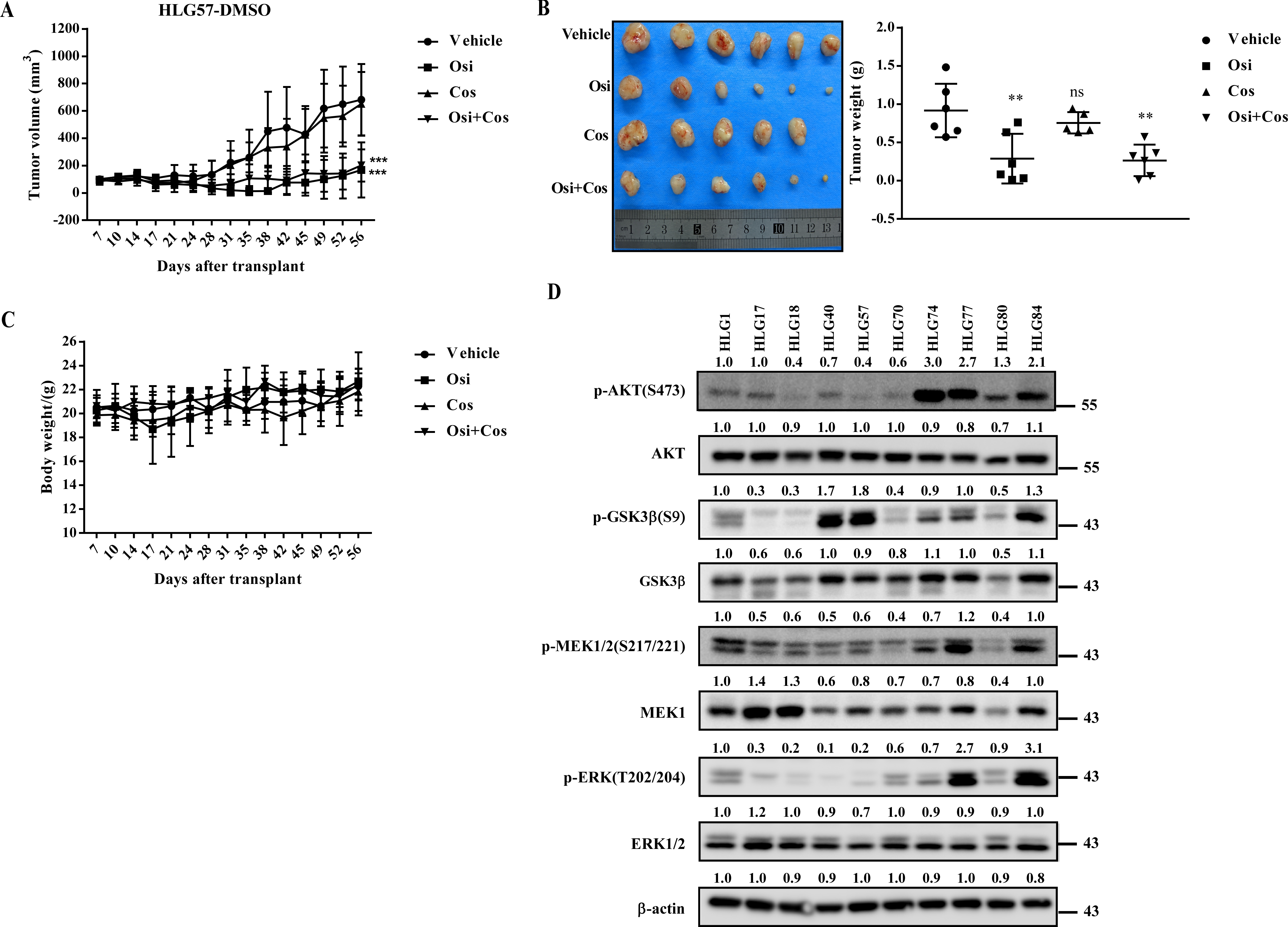

Supplement: Supplementary file 11 — Supplementary Material 11 [file 12943_2022_1662_MOESM11_ESM.jpg]
