# Supplementary material for: Costunolide is a dual inhibitor of MEK1 and AKT1/2 that overcomes osimertinib resistance in lung cancer
Source: Mol Cancer. 2022 Oct 6;21:193. doi: 10.1186/s12943-022-01662-1 (PMC9535870; doi:10.1186/s12943-022-01662-1)
Supplement: Supplementary file 12 — Supplementary Material 12 [file 12943_2022_1662_MOESM12_ESM.docx]

**Supplemental Figure Legends**

**S1. Verification of osimertinib resistance. A**. Parental and resistant cells were cultured with 0, 0.001, 0.01, 0.1 μM concentrations of osimertinib for 1 week. Colony number were calculated using the Image-Pro software. The Y axis shows relative colony number to control group. **B**. Both parental and resistant cells were treated with 0, 1, 2.5, or 5 μM osimertinib concentrations for 24h. All cells in the supernatant and the culture dishes were collected and stained with FITC-Annexin V and PI. The cells were subsequently analyzed by flow cytometer. X axis labeled by FL1-H indicates the PI value. Y axis labeled by FL3-H indicates positive staining of FITC-Annexin V. **C.** Cell lysates from resistant cells and parental cells were loaded to compare phosphorylation of HER2 and HER3. Quantitative analysis of western blotting bands was calculated by Image J software in (C). Data was presented as mean ± SD from 3 independent experiments in (A and B). Unpaired t-test was used in (A and B). **P < 0.01, ***P < 0.001, ****P < 0.001.

**S2. The effect of Costunolide on phosphatase activity of MEK2. A.** Chemical structure of costunolide. B. Effect of costunolide on phosphatase activity of MEK2. 100 ng active MEK2 kinase was pre-incubated with various concentration of costunolide at RT for 15 mins. Next, 200 ng inactive ERK1 and ATP buffer were added and the mixture were incubated at 30 ℃ for 30 mins. Phosphorylatedand total ERK were detected by Western blot. Quantitative analysis of western blotting bands was calculated by Image J software in (B).

**S3. Costunolide binds with MEK1 and AKT1/2. A**. Costunolide binds with MEK1. 200 ng recombinantMEK1 protein was incubated with Sepharose-4B or costunolide-conjugated Sepharose-4B. MEK1 in the pull-down pellet was analyzed by Western blotting. **B**. The same method to assess binding between costunolide and AKT1 or AKT2. **C**. The same method to verify binding between costunolide and c-Kit, Aurora A, ERK1, ERK2, MKK3, MKK6, RSK2 and PI3K.

**S4. Costunolide induces significant cell apoptosis in osimertinib resistant cells**. **A**. Costunolide inhibited osimertinib-resistant cell growth. PC9-DMSO, PC9-Osi, HCC827-DMSO, and HCC827-Osi cells were incubated with 0, 2.5, 5, 10 μM concentrations of costunolide for 48 h followed by MTT assay. Next, cell viability was calculated and compared between each parental cell and resistant cell line. **B**. Parental and resistant cells were treated with 0, 5, 10, 20 μM concentrations of costunolide for 24h. Afterward, all cells were stained by FITC-Annexin V and PI. Apoptotic cells were detected by flow cytometry. **C**. The effect of costunolide treatment on apoptosis markers was determined by Western blotting at 24 h post-costunolide treatment. Date is shown as mean values ± S.D. from 3 independent experiments in (A and B). Quantitative analysis of western blotting bands was calculated by Image J software in (C). Unpaired t-test was used in (A and B). *P< 0.05, **P < 0.01, ***P < 0.001.

**S5. Effects of costunolide compared with MEKi and AKTi. A**. Costunolide has a high safety dose. NL20, SHEE and CCD-18Co cells were seeded and incubated with the indicated concentrations of costunolide for 24h. Cell viability was calculated by MTT assay. **B**. Inhibitory effect on resistant cells. PC9-Osi, HCC827-Osi, H1975-Osi cells were cultured with various concentrations of costunolide, MEKi or AKTi for 48h. Inhibitory effect was indicated by relative cell viability as shown in the Y axis. **C.** Toxicity of MEKi and AKTi. NL20 cells were seeded and incubated with the indicated concentrations of MEKi, AKTi or MEKi plus AKTi for 24h. Cell viability was calculated using MTT assays. Date is shown as mean values ± S.D. from 3 independent experiments in (A, B and C). One-way ANOVA with a multiple comparisons test was used in (A and C). Unpaired t-test was used in (B). ns P > 0.05, ***P < 0.001.

**S6.** **Synergistic inhibitory effect of osimertinib with costunolide on foci formation.** PC9-Osi, HCC827-Osi, and H1975-Osi cells were seeded in 6-well plates and treated with costunolide (2 μM), osimertion (1 μM) or costunolide plus osimertinib for 1 week. Colonies were then stained with crystal violet and counted using the Image J software. Inhibitory effect of each group was compared with the non-treated group. Date is shown as mean values ± S.D. from 3 independent experiments. One-way ANOVA with multiple comparisons analyzes was applied in (A), ****P < 0.0001.

**S7. Establishment of osimertinib resistant PDX model. A**. Schedule used to establish osimertinib resistant PDX model. **B.** Tumor volume**,** tumor shape, and weight from first passage treated mice. **C.** Tumor volume**,** tumor shape, and weight from second passage treated mice. **D.** Tumor volume**,** tumor shape, and weight from third passage treated mice. **E**. Protein lysates from resistant PDX and parental PDX were loaded to compare phosphorylation of EGFR, HER2 and HER3. Quantitative analysis of western blotting bands was calculated by Image J software in (E). One-way ANOVA with post hoc Dunnett’s test was used in (B). *P < 0.05.

**S8. Combination effect of osimertinib and costunolide on osimertinib resistant PDX model.** A. Tumor weight. After mice were sacrificed, tumors were excised and the weight of each tumor was recorded. Data was presented as mean ± SD, (N=5). **B**. Effect on mouse body weight. Body weights of mice in each group were measure twice per week starting 7 days after transplant. Data are presented as mean ± SD, (N=5). One-way ANOVA with post hoc Dunnett’s test was used in (A)., *P < 0.05; **P < 0.01.

**S9. Costunolide exhibited no cytotoxicity *in vivo*. A.** Image of liver, kidney and spleen from sacrificed mice. **B**. H&E staining of tumor, liver, kidney and spleen. **C**. AST activity. Blood from sacrificed mice were collected to separate plasma. The AST activity in each mouse was calculated using an AST assay kit. **D**. ALT activity. Blood from sacrificed mice were collected to separate plasma. The ALT activity in each mouse was calculated using an ALT assay kit. **E.** WBC and RBC measurement. Number of WBC and RBC in blood 24 h after treatment with vehicle or costunolide (20 mg/kg) were measured by hematology analyzer. Data were presented as mean ± SD in (C, D and E) (N=5 mice). One-way ANOVA with a multiple comparisons test was used in (C and D). Wilcoxon single-rank test was used in (E). ns P> 0.05.

**S10. Effect of costunolide on parental PDX model. A.** Inhibitory effect of costunolide (20 mg/kg), osimertinib (10 mg/kg), costunolide plus osimertinib on tumor growth. **B**. Tumor shape and weight. After mice were sacrificed, tumors were excised and tumor weights were recorded. **C**. Effect on mouse body weight. Body weights of mice from each group were measured twice per week starting 7 days after transplant. **D.** Expression levels of AKT, GSK3β, MEK1 and ERK in various PDX tumor tissues were determined by Western blotting. For A-C, data are presented as mean ± SD, (n=5-6). Quantitative analysis of western blotting bands was performed by Image J software in (D). One-way ANOVA with post hoc Dunnett’s test was used in (A), one-way ANOVA with a multiple comparisons test was used in (B), ns P > 0.05, **P < 0.01, ***P < 0.001.
